# Treatment with therapeutic anticoagulation is not associated with immunotherapy response in advanced cancer patients

**DOI:** 10.1186/s12967-021-02712-w

**Published:** 2021-01-30

**Authors:** Paul Johannet, Amelia Sawyers, Nicholas Gulati, Douglas Donnelly, Samuel Kozloff, Yingzhi Qian, Alfredo Floristan, Eva Hernando, Judy Zhong, Iman Osman

**Affiliations:** 1 Department of Medicine, NYU Langone Health, New York, USA; 2 Ronald O. Perelman Department of Dermatology, NYU Langone Health, New York, USA; 3 Department of Population Health, NYU Langone Health, New York, USA; 4 Department of Pathology, NYU Langone Health, New York, USA; 5 Ronald O. Perelman Department of Dermatology, NYU Grossman School of Medicine, 550 First Ave, Smilow 403, New York, NY 10016 USA

## Abstract

**Background:**

Recent preclinical data suggest that there may be therapeutic synergy between immune checkpoint blockade and inhibition of the coagulation cascade. Here, we investigate whether patients who received immune checkpoint inhibitors (ICI) and were on concomitant anticoagulation (AC) experienced better treatment outcomes than individuals not on AC.Affiliation: Kindly confirm if corresponding authors affiliation is identified correctly.The corresponding author's affiliation is correct.

**Methods:**

We studied a cohort of 728 advanced cancer patients who received 948 lines of ICI at NYU (2010–2020). Patients were classified based on whether they did (n = 120) or did not (n = 828) receive therapeutic AC at any point during their treatment with ICI. We investigated the relationship between AC status and multiple clinical endpoints including best overall response (BOR), objective response rate (ORR), disease control rate (DCR), progression free survival (PFS), overall survival (OS), and the incidence of bleeding complications.Affiliations: Journal instruction requires a country for affiliations; however, this is missing in affiliations 1 to 5. Please verify if the provided country is correct and amend if necessary.The country is correct for all affiliations (1 - 5).

**Results:**

Treatment with AC was not associated with significantly different BOR (*P *= 0.80), ORR (*P *=0.60), DCR (*P *=0.77), PFS (*P* = 0.59), or OS (*P *=0.64). Patients who received AC were significantly more likely to suffer a major or clinically relevant minor bleed (*P *= 0.05).

**Conclusion:**

AC does not appear to impact the activity or efficacy of ICI in advanced cancer patients. On the basis of our findings, we caution that there is insufficient evidence to support prospectively evaluating the combination of AC and immunotherapy.

## Background

Immune checkpoint inhibitors (ICI) produce durable clinical response for a subset of advanced cancer patients, but the majority do not experience long-term benefit from treatment [1–3]. Of those who initially respond to ICI, many will later become resistant or suffer disease relapse [4]. Thus, there is an urgent need to delineate the molecular characteristics of innate and acquired resistance to checkpoint blockade. This will not only help optimize patient selection for treatment, but it can also inform rational treatment strategies for overcoming the mechanisms of ICI resistance.

Several recent studies suggest that clotting factors from the coagulation cascade facilitate cancer immune evasion and might therefore promote resistance to ICI [5, 6]. Metelli et al. provide mechanistic evidence that thrombin cleaves glycoprotein A repetitions predominant (GARP), which in turn mediates the release of transforming growth factor-ß (TGF-ß), a cytokine that is known to downregulate CD8^+^ T cells, upregulate CD4^+^ T cells, and reduce immune cell infiltration of tumors by inducing collagen and fibroblast barriers [5]. Graf et al. found that myeloid cell synthesized factor Xa promotes tumor immune evasion [6]. Together, these studies also showed that concomitant treatment with anticoagulants plus immunotherapy led to synergistic attenuation of tumor growth and better survival in mouse models of colon cancer and fibrosarcoma. These data are consistent with previous reports that hemostatic factors impede the innate anticancer immune response by interfering with natural killer cell activity [7, 8].

To our knowledge, these robust preclinical findings have not been confirmed in the clinical setting. On the contrary, one group recently showed that AC was not associated with better progression free survival (PFS) or overall survival (OS) in non-small cell lung cancer (NSCLC) patients treated with PD-1 or PD-L1 blockade [9]. However, this study only analyzed 47 patients with one cancer type. Since treatment with AC is associated with increased risk of bleeding in cancer patients, whose disease already predisposes them to bleeding complications, the possibility of therapeutic synergy between AC and ICI warrants comprehensive evaluation in advance of prospective testing [10, 11]. In this study, we investigated whether treatment with AC is associated with ICI response in a large cohort of advanced cancer patients.

## Methods

### Patient population

Clinicodemographic data were extracted from two IRB-approved databases at NYU Langone Health (IRB #10362 and IRB #S16-00122). All patients provided written informed consent to participate in this study. The study cohort consisted of patients with stage III or IV cancer who were treated with one or more lines of immunotherapy between 2010 and 2020. We searched the electronic medical record for patients who received any of the following anticoagulants while simultaneously on ICI: direct thrombin inhibitors (dabigatran), factor Xa inhibitors (apixaban and rivaroxaban), heparin products (enoxaparin and heparin), and vitamin K antagonists (warfarin). AC status during each ICI treatment line was categorized as “on therapeutic AC” or “not on therapeutic AC.” We excluded prophylactic AC from the analyses, which was defined according to institutional protocol as heparin 5000 units every 8–12 h and lovenox 30–40 mg every 24 h. Clinical decisions regarding the prescription of therapeutic AC were made independent of this study.

### Clinical outcomes

We evaluated the relationship between AC status and the following clinical outcomes: best overall response (BOR), objective response rate (ORR), disease control rate (DCR), progression free survival (PFS), and overall survival (OS). Response was classified as complete response (CR), partial response (PR), stable disease (SD), or progressive disease (PD) according to the revised Response Evaluation Criteria in Solid Tumors (RECIST) guidelines (version 1.1). ORR was the proportion of patients with CR or PR. DCR was the proportion of patients with CR, PR or SD. PFS was the time from treatment start until disease progression or death from disease. OS was the time from treatment start until death from any cause. The incidence of bleeding complications while on immunotherapy was analyzed for each ICI treatment line. Bleeding was categorized as major and clinically relevant minor according to the criteria outlined by the International Society on Thrombosis and Haemostasis [12, 13]. Major bleeding included fatal bleeding, symptomatic bleeding in a critical organ, and/or bleeding causing a decrease of hemoglobin level by 2 g/dL or more, or leading to a transfusion of two or more units of whole blood or red cells. Clinically relevant minor bleeds did not meet criteria for a major bleed but prompted a clinical response including hospital admission, increased level of care, or medical intervention by a healthcare professional such as a change in antithrombotic therapy.

### Statistical analyses

Associations between AC status and BOR, ORR, DCR, and bleeding outcomes were assessed using the Chi-square test. The 95% confidence intervals were calculated using the Clopper-Pearson method. The association between bleeding outcomes and different anticoagulation categories was assessed using the Fisher’s exact test. We generated Kaplan–Meier survival curves to determine the association between AC status and PFS and OS and compared them using the log-rank test. We then performed multivariable Cox proportional hazards regression analyses by stratifying by cancer type and adjusting for age, sex, disease stage, Eastern Cooperative Oncology Group (ECOG) performance status, treatment regimen, treatment line, anticoagulant category, and whether immunotherapy was given alone or in combination with other pharmacologic agents. All analyses were performed using R version 4.0.2. Statistical tests were 2-sided and *P *≤ 0.05 was considered significant.

## Results

The clinical and demographic characteristics of the study cohort are shown in Table 1. There was a total of 728 patients who received 948 lines of immunotherapy. Therapeutic AC was prescribed during 120 (12.7%) of the treatment lines. Patients were treated with apixaban (n = 36), dabigatran (n = 12), enoxaparin (n = 27), rivaroxaban (n = 24), or warfarin (n = 21) (Additional file 1: Table S1). The most common indications for therapeutic AC were arrhythmias (n = 47), pulmonary embolism (n = 37), and deep vein thrombosis (n = 36). In total, 25/27 (92.6%) patients were prescribed enoxaparin for deep vein thrombosis, pulmonary embolism, or both, as compared to only 8/21 (38.1%) patients on warfarin, 2/12 (16.7%) patients on direct thrombin inhibitors, and 29/60 (48.3%) patients on factor Xa inhibitors (Additional file 1: Table 2). Patients who received AC were significantly older than their counterparts (70.5 versus 62.0 years old; p < 0.0001). Patients who received AC also had worse ECOG performance status (p = 0.0001).

**Table 1:** Clinical and demographic characteristics of the cohort

	Not on Anticoagulation	On anticoagulation	*p* value
Total number of patients	828	120	
Age (years)			< 0.0001
Mean (SD)	62.0 (13.9)	70.5 (11.6)	
Range	20.4–94.2	31.7–94.6	
Sex			0.50
Female	372 (44.9)	50 (41.7)	
Male	456 (55.1)	70 (58.3)	
ECOG			0.0001
0	447 (54.0)	43 (35.8)	
1	290 (35.0)	54 (45.0)	
≥ 2	60 (7.2)	20 (16.7)	
Unknown	31 (3.7)	3 (2.5)	
Primary tumor			< 0.0001
Melanoma	417 (50.4)	48 (40.0)	
Lung	139 (16.8)	40 (33.3)	
Other	272 (32.9)	32 (26.7)	
Stage			0.56
III	149 (18.0)	19 (15.8)	
IV	679 (82.0)	101 (84.2)	
Treatment regimen			0.002
Anti-CTLA-4	166 (20.0)	9 (7.4)	
Anti-PD-1 or Anti-PD-L1	497 (60.0)	93 (77.5)	
Anti-PD-1 + Anti-CTLA-4	165 (19.9)	18 (15.0)	
Treatment line			0.75
First	632 (76.3)	90 (75.0)	
Non-first	196 (23.7)	30 (25.0)	
Immunotherapy alone or in combination with other therapy			0.19
Alone	632 (76.3)	85 (70.8)	
With other therapy	196 (23.7)	35 (29.2)	
Alive status			0.11
Alive	381 (46.0)	46 (38.3)	
Deceased	447 (54.0)	74 (61.7)

**Table 2:** Response to immunotherapy based on anticoagulation status

Outcome	Not on anticoagulation (n = 828)	On anticoagulation (n = 120)	p-value
Best overall response—no. (%)			0.80
Complete response (CR)	129 (15.6)	14 (11.7)	
Partial response (PR)	140 (16.9)	23 (19.2)	
Stable disease (SD)	144 (17.4)	22 (18.3)	
Progressive disease (PD)	395 (47.7)	60 (50.0)	
Could not be evaluated	10 (1.2)	1 (0.8)	
Objective response rate^a^			0.60
No. (%)	279 (33.6)	37 (30.8)	
95% CI^b^	30.4–37.0	22.7–40.0	
Disease control rate^c^			0.77
No. (%)	423 (51.1)	59 (49.2)	
95% CI^b^	47.6–54.5	40.0–58.4	

^a^Objective response rate was defined as the percentage of patients who had CR or PR

^b^The 95% confidence intervals were calculated using the Clopper-Pearson method

^c^Disease control rate was defined as the percentage of patients who had CR, PR or SD

Table 2 shows the BOR, ORR, and DCR of patients based on their AC status. There was no significant difference in the BOR of patients who did or did not receive AC (*P *= 0.80). In line with this finding, there was also no significant difference in their ORR (*P *=0.60) or DCR (*P *=0.77). Patients who received AC had an ORR of 30.8% (95% CI 22.7–40.0) while those not on AC had an ORR of 33.6% (95% CI 30.4–37.0). The DCR of patients on AC was 49.2% (95% CI 40.0–58.4) and those not on AC had a DCR of 51.1% (95% CI 47.6–54.5).

**Fig. 1:**
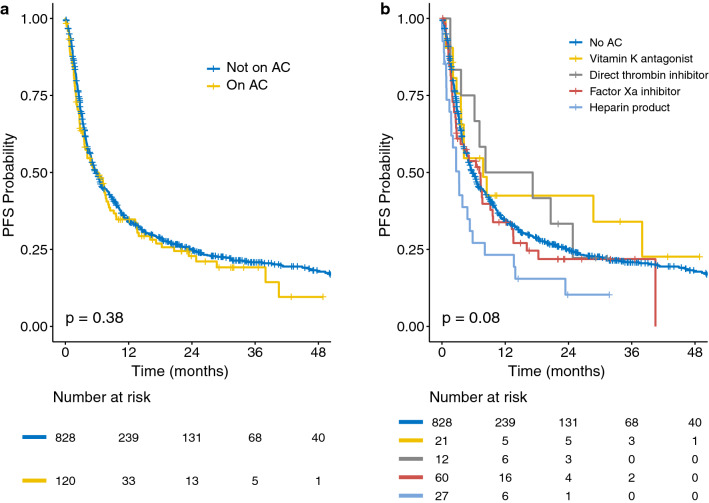
Kaplan-Meier curves show progression free survival (PFS) in patients based on their anticoagulation (AC) status. PFS in **a** patients stratified based on whether they did or did not receive AC while being treated with immunotherapy, and **b** patients stratified based on the anticoagulant with which they were treated

There was no significant association between PFS and AC status in univariable analyses (P = 0.38). This was also the case when analyzing PFS based on the category of anticoagulant (*P *=0.08) (Fig. 1). In multivariable analyses, after adjusting for potential confounders, there was still no significant association between PFS and AC status (HR: 1.07; 95% CI 0.85–1.34; *P* = 0.59) (Additional file 1: Table S3). When analyzing outcomes by category of anticoagulant, the multivariable analysis showed that patients on enoxaparin had significantly worse PFS (HR: 2.11; 95% CI 1.46–3.06; *P* < 0.001) (Additional file 1: Table S4). There was no significant difference in PFS for patients on warfarin (*P *=0.69), direct thrombin inhibitors (*P *=0.56), or factor Xa inhibitors (*P* = 0.29).

**Fig. 2:**
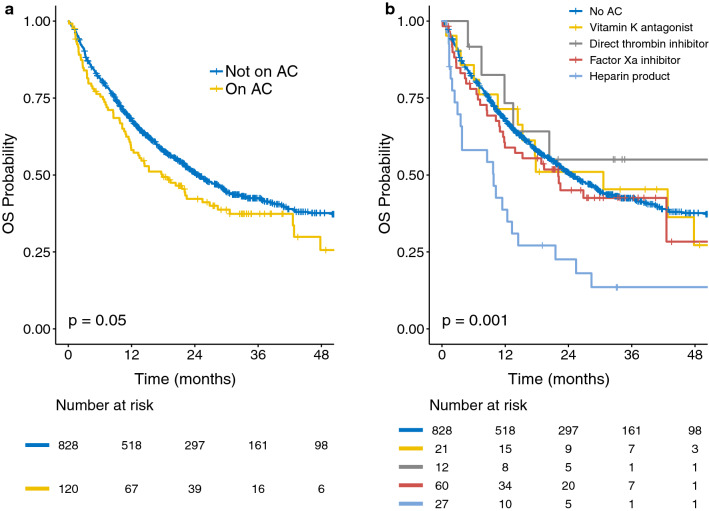
Kaplan-Meier curves show overall survival (OS) in patients based on their anticoagulation (AC) status. OS in **a** patients stratified based on whether they did or did not receive AC while being treated with immunotherapy, and **b** patients stratified based on the anticoagulant with which they were treated

In univariable analyses, patients on AC had significantly worse OS than those not on AC (*P *= 0.05). However, this relationship was not significant in multivariable analyses (HR: 1.08; 95% CI 0.79–1.46; *P *=0.64) (Additional file 1: Table S3). In univariable analyses, there was a significant association between OS and the category of anticoagulant prescribed (*P *=0.001) (Fig. 2). The multivariable analysis showed that patients on enoxaparin had significantly worse OS (HR: 2.86; 95% CI 1.91–4.30; *P* < 0.001) (Additional file 1: Table S4). In contrast, compared to patients not on AC during ICI, the patients on warfarin, direct thrombin inhibitors, and factor Xa inhibitors did not have significantly different OS (*P *=0.80, *P *=0.57, and *P *=0.31, respectively).

Patients on therapeutic AC during immunotherapy were significantly more likely to suffer a major or clinically relevant minor bleed at some point during their ICI treatment compared to patients not on AC (*P *=0.05) (Table 3). A total of 4 (3.3%) anticoagulated patients experienced major bleeds whereas 19 (2.3%) non-AC patients suffered a major bleed (*P *= 0.71). There were 6 (5.0%) AC patients and 13 (1.6%) non-AC patients who had a clinically relevant minor bleed (*P *= 0.03). There was no significant difference in the incidence of major or clinically relevant minor bleeding between different categories of AC (*P *=0.27) (Additional file 1: Table S5).

**Table 3:** Summary of bleeding complications during treatment with immunotherapy

Outcome	Not on anticoagulation (n = 828)	On anticoagulation (n = 120)	p-value
Bleeding outcomes^a^			0.05
No. (%)	32 (3.9)	10 (8.3)	
95% CI^b^	2.7–5.4	4.1–14.8	

^a^Bleeding outcomes included major and clinically relevant minor bleeds

^b^ The 95% confidence intervals were calculated using the Clopper-Pearson method

## Discussion

Recent preclinical data suggest that clotting factors promote cancer immune evasion, and that inhibition of the coagulation cascade subsequently improves response to immunotherapy [5, 6]. On the basis of these findings, one possible approach to augmenting anticancer immune activity would be treating patients with concomitant AC and ICI. In this study, we investigated the validity of this therapeutic strategy in a cohort of advanced cancer patients with multiple different primary malignancies. We observed that patients who received AC while simultaneously on ICI did not experience significantly different treatment response or survival. However, the patients on therapeutic AC were more likely to suffer from bleeding complications. Our study adds to a limited body of clinical research on the association between AC and ICI response. To our knowledge, only one other recent report showed that PFS and OS were not associated with AC status in a cohort of 47 NSCLC patients [9]. In fact, Nichetti et al. (2020) found that there was a trend towards worse PFS for patients on AC, although this can in part be explained by their worse ECOG grades. The patients on AC in our study also had worse performance status than their counterparts, which is a reflection of the severity of their disease burden, and, in parallel, the need for improved therapeutic strategies. Even though these individuals were older and had worse performance status, there were no significant differences in immunotherapy treatment outcomes after adjusting for these potential confounders in multivariable analyses, which suggests that AC is not associated with ICI response.

Importantly, we determined that there was no individual class of anticoagulant which was associated with better survival outcomes compared to patients not on AC. Although preclinical studies in mouse models demonstrated synergy between ICI and dabigatran and rivaroxaban, our data showed that patients on direct thrombin inhibitors and factor Xa inhibitors had no statistically significant difference in PFS or OS [5, 6]. Interestingly, we observed that patients on enoxaparin had significantly worse PFS and OS compared to patients not on AC as well as to patients on different classes of anticoagulants. This held true in the multivariable analyses, which suggests that patients on enoxaparin did not do worse simply because of established confounders such as age and ECOG grade. Enoxaparin has been the anticoagulant most commonly prescribed for cancer patients with venous thromboembolism, although emerging evidence suggests that factor Xa inhibitors are equally efficacious as heparin products [14–16]. In line with this, we found that the vast majority of patients prescribed enoxaparin in our cohort (92.6%) received it for the treatment of venous thromboembolism. In contrast, only 48.3% of patients on factor Xa inhibitors, 38.1% of patients on warfarin, and 16.7% on direct thrombin inhibitors had VTE. The difference in overall disease burden, as reflected by the presence or absence of VTE, is most likely what accounts for differences in PFS and OS. Thus, for patients who require therapeutic AC, the class of AC should not be selected based on whether or not the patient is receiving immunotherapy.

Current experimental evidence in support of combining AC with ICI comes from mouse models of colon cancer and fibrosarcoma [5, 6]. Although our cohort included 26 patients with mesothelial or soft tissue malignancies, there was only 1 patient with fibrosarcoma, and there were 0 patients with colon cancer. Given the robustness of the preclinical data, it will be important for future studies to determine the association between AC status and immunotherapy outcomes in patients with these cancer types. However, the available data do not support the prescription of anticoagulants to patients on immunotherapy in the absence of standard indications for AC. We caution the scientific community that there is insufficient evidence to support prospective studies of AC in combination with ICI. Further retrospective analyses will need to provide strong evidence that inhibition of the coagulation cascade augments ICI response in order to justify the increased risk of bleeding that comes with prescribing anticoagulants.

## Conclusions

As immune checkpoint inhibitors are increasingly used to treat multiple different malignancies, it is imperative to understand whether and to what degree treatment outcomes are affected by patients’ concomitant medications. It is equally important to identify factors that associate with improved immunotherapy response. Several recent preclinical studies report that there might be a synergistic effect between immune checkpoint inhibitors and anticoagulants. However, our data suggest that anticoagulants do not impact the efficacy of immune checkpoint inhibitors, but are associated with a significantly increased risk of bleeding. These findings should guide the allocation of resources towards investigating alternative means for augmenting immunotherapy response. Future research should also explore the interaction between immune checkpoint inhibitors and other commonly prescribed medications such as antiplatelet agents.

## Supplementary information

**Additional file 1.** Additional Tables.

## Data Availability

Data is available from the authors upon reasonable request, but may require data transfer agreements. No personal health information will be shared.

## References

[1] Hodi FS (2010). Improved survival with ipilimumab in patients with metastatic melanoma. N Engl J Med.

[2] Reck M (2016). Pembrolizumab versus chemotherapy for PD-L1-positive non-small cell lung cancer. N Engl J Med.

[3] Motzer RJ (2018). Nivolumab plus ipilimumab versus sunitinib in advanced renal-cell carcinoma. N Engl J Med.

[4] Sharma P (2017). Primary, adaptive and acquired resistance to cancer immunotherapy. Cell.

[5] Metelli A (2020). Thrombin contributes to cancer immune evasion via proteolysis of platelet-bound GARP to activate LTGF-ß. Sci Transl Med..

[6] Graf C (2019). Myeloid cell-synthesized coagulation factor X dampens antitumor immunity. Sci Immunol..

[7] Palumbo JS (2007). Tumor cell–associated tissue factor and circulating hemostatic factors cooperate to increase metastatic potential through natural killer cell–dependent and–independent mechanisms. Blood.

[8] Palumbo JS (2005). Platelets and fibrin(ogen) increase metastatic potential by impeding natural killer cell-mediated elimination of tumor cells. Blood.

[9] Nichetti F (2019). Is there an interplay between immune checkpoint inhibitors, thromboprophylactic treatments and thromboembolic events? Mechanisms and impact in non-small cell lung cancer patients. Cancers..

[10] Angelini DE (2019). Bleeding incidence and risk factors among cancer patients treated with anticoagulation. Am J Hematol.

[11] Johnston C, Rich SE (2018). Bleeding in cancer patients and its treatment: a review. Ann Palliat Med..

[12] Schulman S (2005). Definition of major bleeding in clinical investigations of antihemostatic medicinal products in non-surgical patients. J Thromb Haemost.

[13] Kaatz S (2015). Definition of clinically relevant non-major bleeding in studies of anticoagulants in atrial fibrillation and venous thromboembolic disease in non-surgical patients: communication from the SSC of the ISTH. J Thromb Haemost.

[14] Connors A (2020). Apixaban for the treatment of venous thromboembolism associated with cancer. N Engl J Med.

[15] Raskob GE (2018). Edoxaban for the treatment of cancer-associated venous thromboembolism. N Engl J Med.

[16] Lyman GH (2015). Venous thromboembolism prophylaxis and treatment in patients with cancer: American society of clinical oncology clinical practice guideline update 2014. J Clin Oncol.

