# Supplementary material for: Treatment with therapeutic anticoagulation is not associated with immunotherapy response in advanced cancer patients
Source: J Transl Med. 2021 Jan 30;19:47. doi: 10.1186/s12967-021-02712-w (PMC7847556; doi:10.1186/s12967-021-02712-w)
Supplement: Supplementary file 1 — Additional file 1. Additional Tables. [file 12967_2021_2712_MOESM1_ESM.docx]

Table S1. Summary of the prescribed anticoagulants.

| **Anticoagulant** | **N (%)** |
| --- | --- |
| Direct thrombin inhibitors |  |
| Dabigatran (Pradaxa) | 12 (10.0) |
| Factor Xa inhibitors |  |
| Apixaban (Eliquis) | 36 (30.0) |
| Rivaroxaban (Xarelto) | 24 (20.0) |
| Heparin products |  |
| Enoxaparin (Lovenox) | 27 (22.5) |
| Vitamin K antagonists |  |
| Warfarin (Coumadin) | 21 (17.5) |

Table S2. Summary of the indications for therapeutic anticoagulation.

| **Anticoagulant category** | **Indication [N (%)]** | | | | |
| --- | --- | --- | --- | --- | --- |
|  | **VTE** | **Arrhythmia** | **CVA** | **Valve replacement** | **Other/Unknown** |
| Direct thrombin inhibitor | 2 (16.7%) | 10 (83.3%) | 0 (0%) | 0 (0%) | 0 (0%) |
| Factor Xa inhibitor | 29 (48.3%) | 25 (41.7%) | 2 (3.3%) | 0 (0%) | 7 (11.7%) |
| Heparin products | 25 (92.6%) | 2 (7.4%) | 0 (0%) | 0 (0%) | 1 (3.7%) |
| Vitamin K antagonist | 8 (38.1%) | 10 (47.6%) | 0 (0%) | 3 (14.3%) | 0 (0%) |

Venous thromboembolism (VTE) includes deep vein thrombus (DVT), pulmonary embolism (PE), or both. CVA refers to cerebrovascular accidents. The sum total within each indication does not always equal number of patients in the corresponding AC category because some patients received AC for multiple indications.

Table S3. Results of the multivariable Cox proportional-hazards regression analysis with patients stratified by anticoagulation status.

| **Variable** | **PFS** | | **OS** | |
| --- | --- | --- | --- | --- |
|  | **HR [95% CI]** | **p-value** | **HR [95% CI]** | **p-value** |
| On AC during ICI (ref: not on AC) | 1.07 [0.85, 1.34] | 0.59 | 1.08 [0.79, 1.46] | 0.64 |
| ECOG (ref: >1) |  |  |  |  |
| ECOG = 0 | 0.44 [0.31, 0.62] | <0.001 | 0.30 [0.21, 0.45] | <0.001 |
| ECOG = 1 | 0.78 [0.57, 1.07] | 0.12 | 0.68 [0.49, 0.96] | 0.03 |
| Stage | 1.34 [1.07, 1.67] | 0.01 | 1.75 [1.31, 2.33] | <0.001 |
| Age | 0.99 [0.99, 1.00] | 0.08 | 1.00 [0.99, 1.01] | 0.67 |
| Sex (male) | 1.06 [0.89, 1.26] | 0.52 | 1.28 [1.01, 1.62] | 0.04 |
| Treatment line = not first line | 1.77 [1.45, 2.16] | <0.001 | 1.65 [1.32, 2.06] | <0.001 |
| Anti-CTLA-4 plus anti-PD-1 (ref: anti-CTLA-4) | 0.47 [0.36, 0.61] | <0.001 | 0.47 [0.33, 0.66] | <0.001 |
| Anti-PD-1 (ref: anti-CTLA-4) | 0.40 [0.31, 0.52] | <0.001 | 0.43 [0.32, 0.58] | <0.001 |
| Anti-PD-L1 (ref: anti-CTLA-4) | 0.38 [0.23, 0.63] | <0.001 | 0.41 [0.23, 0.74] | 0.003 |
| ICI plus chemotherapy (ref: ICI alone) | 1.01 [0.81, 1.27] | 0.90 | 0.96 [0.73, 1.27] | 0.80 |

Table S4. Results of the multivariable Cox-proportional hazards regression analysis with patients stratified by category of anticoagulation.

| **Variable** | **PFS** | | **OS** | |
| --- | --- | --- | --- | --- |
|  | **HR [95% CI]** | **p-value** | **HR [95% CI]** | **p-value** |
| Category of AC (ref: not on AC) |  |  |  |  |
| Direct thrombin inhibitor | 1.18 [0.68, 2.05] | 0.56 | 0.76 [0.30, 1.94] | 0.57 |
| Factor Xa inhibitor | 0.84 [0.60, 1.16] | 0.29 | 0.81 [0.54, 1.22] | 0.31 |
| Heparin product | 2.11 [1.46, 3.06] | <0.001 | 2.86 [1.91, 4.30] | <0.001 |
| Vitamin K antagonist | 0.89 [0.50, 1.57] | 0.69 | 0.91 [0.42, 1.97] | 0.80 |
| ECOG (ref: >1) |  |  |  |  |
| ECOG = 0 | 0.42 [0.29, 0.59] | <0.001 | 0.29 [0.20, 0.42] | <0.001 |
| ECOG = 1 | 0.75 [0.55, 1.03] | 0.08 | 0.66 [0.47, 0.92] | 0.02 |
| Stage | 1.36 [1.09, 1.71] | 0.008 | 1.78 [1.33, 2.38] | <0.001 |
| Age | 0.99 [0.99, 1.00] | 0.11 | 1.00 [0.99, 1.01] | 0.49 |
| Sex (male) | 1.06 [0.89, 1.26] | 0.53 | 1.29 [1.02, 1.63] | 0.04 |
| Treatment line (ref: first line | 1.77 [1.45, 2.17] | <0.001 | 1.64 [1.31, 2.05] | <0.001 |
| Anti-CTLA-4 plus anti-PD-1 (ref: anti-CTLA-4) | 0.47 [0.36, 0.61] | <0.001 | 0.47 [0.33, 0.66] | <0.001 |
| Anti-PD-1 (ref: anti-CTLA-4) | 0.40 [0.31, 0.52] | <0.001 | 0.43 [0.32, 0.58] | <0.001 |
| Anti-PD-L1 (ref: anti-CTLA-4) | 0.39 [0.23, 0.63] | <0.001 | 0.43 [0.24, 0.77] | 0.005 |
| ICI plus chemotherapy (ref: ICI alone) | 1.00 [0.80, 1.26] | 0.98 | 0.95 [0.72, 1.25] | 0.70 |

Table S5. Summary of bleeding complications according to AC category

| Variable | Bleed while on ICI* (n=10) | No bleed (n=110) | p-value^#^ |
| --- | --- | --- | --- |
| Anticoagulation category |  |  | 0.27 |
| Direct thrombin inhibitor | 0 (0%) | 12 (10.9%) |  |
| Factor Xa inhibitor | 8 (80.0%) | 52 (47.3%) |  |
| Heparin products | 2 (20.0%) | 25 (22.7%) |  |
| Vitamin K antagonist | 0 (0%) | 21 (19.1%) |  |

*Bleeding outcomes include major and clinically relevant minor bleeds

^#^P-value determined using Fisher’s exact test
